# Draft Genome Sequences of Three Strains of *Bacteroides pyogenes* Isolated from a Cat and Swine

**DOI:** 10.1128/genomeA.01242-13

**Published:** 2014-01-30

**Authors:** Mitsuo Sakamoto, Kenshiro Oshima, Wataru Suda, Keiko Kitamura, Toshiya Iida, Masahira Hattori, Moriya Ohkuma

**Affiliations:** a Microbe Division/Japan Collection of Microorganisms, RIKEN BioResource Center, Tsukuba, Ibaraki, Japan; b Center for Omics and Bioinformatics, Graduate School of Frontier Sciences, University of Tokyo, Kashiwa, Chiba, Japan

## Abstract

Here, we report the draft genome sequences of *Bacteroides pyogenes* JCM 6294^T^, JCM 6292, and JCM 10003, which were isolated from a cat and swine and were recently classified into a single species, *B. pyogenes*. Comparative analyses of these genomes revealed the diversification of *B. pyogenes* strains isolated from different animals.

## GENOME ANNOUNCEMENT

*Bacteroides pyogenes* JCM 6294^T^ and *Bacteroides suis* JCM 6292^T^ were isolated from pigs (1), and *Bacteroides tectus* JCM 10003^T^ was isolated from a cat (2). It was previously reported that the sequence similarities between *B. pyogenes* JCM 6294^T^ and *B. suis* JCM 6292^T^ are 100% for the *hsp60* gene and 100% for the 16S rRNA gene (3). It was also found that the similarities of *B. tectus* JCM 10003^T^ and *B. pyogenes* JCM 6294^T^ or *B. suis* JCM 6292^T^ are 99.6% for the *hsp60* gene and 98.7% for the 16S rRNA gene. Consequently, *B. suis* and *B. tectus* were found to be heterotypic synonyms of *B. pyogenes* based on the DNA-DNA relatedness values (>70%) (3). Therefore, the genome sequences of the three strains are expected to provide new insights into this species concept.

Chromosomal DNAs were extracted from *B. pyogenes* JCM 6294^T^, JCM 6292, and JCM 10003 using a Genomic-tip 100/G (Qiagen). Whole-genome sequencing was performed using the Ion Torrent PGM systems. Low-quality and short reads were removed, and the remaining 542,315, 626,088, and 588,902 reads were assembled into 107, 112, and 95 contigs, respectively, using Newbler version 2.8 (454 Life Sciences). The draft genome sequences resulted in final assemblies of 3,404,843 bp (32.9× redundancy, a G+C content of 45.7%, and an N_50_ of 70,521 bp), 3,405,221 bp (38.1× redundancy, a G+C content of 45.7%, and an N_50_ of 63,669 bp), and 3,379,354 bp (36.8× redundancy, a G+C content of 46.2%, and an N_50_ of 81,559 bp) for *B. pyogenes* JCM 6294^T^, JCM 6292, and JCM 10003, respectively. The draft genomes were annotated by the RAST server (4) using Glimmer3 (5). The *B. pyogenes* JCM 6294^T^ genome contains 3,379 protein-coding sequences (CDSs), a gene density of 86.8%, three rRNAs, and 61 tRNA sequences. JCM 6292 contains 3,340 CDSs, a gene density of 86.8%, three rRNAs, and 60 tRNA sequences. JCM 10003 contains 3,438 CDSs, a gene density of 86.1%, three rRNAs, and 55 tRNA sequences. According to the Genome Blast Distance Phylogeny (GBDP)-based DNA-DNA hybridization (DDH) prediction (6), the pair of *B. pyogenes* JCM 6294^T^ and JCM 6292 genome sequences show a 100% DDH value calculated by the Genome-to-Genome Distance Calculator (GGDC) Web server (GGDC 2.0 [http://ggdc.dsmz.de/distcalc2.php]). On the other hand, the pairs of JCM 10003 and *B. pyogenes* JCM 6294^T^ or JCM 6292 show a 64.7% DDH value, which is considered a subspecies. A BLAST dot plot analysis comparison in the SEED Viewer (http://www.theseed.org) indicated a high degree of genome rearrangements between JCM 10003 and the *B. pyogenes* JCM 6294^T^ or JCM 6292 genome sequences. JCM 10003 contains large numbers of conjugative transposon proteins. This might have contributed to the rearrangement of the genomic structure and led to the diversification of *B. pyogenes*. Further genome analysis will improve our understanding of this species.

### Nucleotide sequence accession numbers.

The draft genome sequences of *B. pyogenes* JCM 6294^T^, JCM 6292, and JCM 10003 have been deposited in DDBJ/EMBL/GenBank under the accession no. BAIR00000000, BAIQ00000000, and BAIU00000000, respectively.
